# Supplementary material for: MetaRibo-Seq measures translation in microbiomes
Source: Nat Commun. 2020 Jun 29;11:3268. doi: 10.1038/s41467-020-17081-z (PMC7324362; doi:10.1038/s41467-020-17081-z)
Supplement: Supplementary file 10 — Supplementary Data 7 [file 41467_2020_17081_MOESM10_ESM.zip › File2/Confidence_High_Taxonomy/173718_out.krona.html]

Javascript must be enabled to view this page.

members
magnitude
magnitudeUnassigned
count
unassigned
taxon
rank

173718\_out

2

2
2
superkingdom

phylum
1239
2

class
186801
2

2
186802
order

186806
1
family

genus
1730
1


SRS065504\_contig\_number\_39448
1
2292352
species

species
1950822

SRS143466\_contig\_number\_contig-100\_21860.74528
1
